# Measurement properties of the Inflammatory Rasch-built Overall Disability Scale (I-RODS) in patients with Guillain–Barré syndrome

**DOI:** 10.1007/s00415-026-13684-6

**Published:** 2026-02-25

**Authors:** Farah Pelouto, Nowshin Papri, Juanita A. Haagsma, David R. Cornblath, Eduardo Nobile-Orazio, Eveline J. A. Wiegers, Thomas Harbo, Yusuf A. Rajabally, Pieter A. van Doorn, Kenneth C. Gorson, Caroline B. Terwee, Bart C. Jacobs, Mike C. Horton, the IGOS Consortium, G. Antonini, S. Arends, S. Attarian, F. A. Barroso, K. Bateman, L. Benedetti, B. van den Berg, P. Y. K. van den Bergh, D. Binda, J. Bürmann, C. Casasnovas, G. Cavaletti, L. L. Cejas, D. R. Cornblath, E. Dardiotis, A. Davidson, A. Y. Doets, P. A. van Doorn, F. Eftimov, T. E. Feasby, G. Galassi, J. M. Goldstein, K. C. Gorson, V. Granit, G. Gutiérrez-Gutiérrez, T. Harbo, J. K. L. Holt, S. T. Hsieh, B. Islam, Z. Islam, B. C. Jacobs, H. D. Katzberg, L. C. de Koning, A. J. van der Kooi, J. C. H. M. Kramers, K. Kuitwaard, S. Kuwabara, H. C. Lehmann, S. E. Leonhard, S. T. Lucy, L. W. G. Luijten, G. A. Marfia, M. M. Martínez- Martínez, L. Martín-Aguilar, G. Mataluni, J. A. L. Miller, Q. D. Mohammad, M. Morales de la Prida, E. Nobile-Orazio, R. J. Nowak, J. Pardo, F. Pelouto, Y. Péréon, L. Querol, A. Rajpar, Y. A. Rajabally, S. Rinaldi, P. Ripellino, J. Roodbol, J. P. A. Samijn, A. Schenone, M. J. Sedano Tous, N. Shahrizaila, K. A. Sheikh, S. H. Sindrup, R. C. M. Thomma, D. Tigner, J. C. Verboon, F. H. Vermeij, M. V. Vytopil, W. Waheed, C. Walgaard, Y. Z. Wang, E. J. A. Wiegers, P. W. Wirtz, C. Wilkerson, M. van Woerkom

**Affiliations:** 1 Department of Neurology, Erasmus MC, University Medical Center Rotterdam, Rotterdam, The Netherlands; 2 Laboratory of Gut-Brain Axis, Icddr,b, Dhaka, Bangladesh; 3 Department of Public Health, Erasmus MC, University Medical Center Rotterdam, Rotterdam, The Netherlands; 4 Department of Neurology, Johns Hopkins University, Baltimore, USA; 5 Department of Translational Medicine, University of Milan IRCCS Humanitas Clinical Institute, Milan, Italy; 6 Department of Neurology, Aarhus University Hospital, Aarhus, Denmark; 7 Aston Medical School, Aston University, Birmingham, UK; 8 Neurology, Tufts University School of Medicine, Boston, USA; 9 Department of Epidemiology and Data Science, Amsterdam UMC, Vrije Universiteit Amsterdam, Amsterdam, The Netherlands; 10 Amsterdam Public Health Research Institute, Methodology, Amsterdam, The Netherlands; 11 Department of Immunology, Erasmus MC, University Medical Center Rotterdam, Rotterdam, The Netherlands; 12 Leeds Psychometric Laboratory for Health Sciences, University of Leeds, Leeds, UK

**Keywords:** Guillain–Barré syndrome (GBS), Inflammatory Rasch-built Overall Disability Scale (I-RODS), Measurement properties, Patient-reported outcome measure (PROM)

## Abstract

Guillain–Barré syndrome (GBS) is an acute immune-mediated polyneuropathy with a high disease impact, even after good clinical recovery. The Inflammatory Rasch-built Overall Disability Scale (I-RODS) is a Patient-Reported Outcome Measure (PROM) developed for patients with immune-mediated neuropathies that measures limitations in daily activities and social participation. It consists of 24 items, scored from 0–48. The present study aimed to validate the measurement properties of the I-RODS in patients with GBS included in the prospective International GBS Outcome Study (IGOS). The current study focussed on structural validity, cross-cultural validity, internal consistency, and construct validity of I-RODS using Rasch-based methods. The study was conducted in 1226 patients diagnosed with GBS with a median I-RODS score of 28 (IQR 10–41) 4 weeks after inclusion into IGOS. Rasch analyses revealed adequate internal consistency (PSI = 0.95; *α* = 0.98) and sufficient construct validity, indicated by strong correlations (*R* = − 0.91 to − 0.77). Targeting was acceptable, although there was a skew towards the floor (10.1%) and ceiling (9.4%). However, the I-RODS showed poor unidimensionality (12.1% CI 10.7–13.5%) and poor overall fit to the Rasch model. Category thresholds were correctly ordered. Misfit was found in two items. Additionally, 15 of 276 item pairs showed local dependency. While no differential item functioning (DIF) was evident for age or sex, significant DIF by geographic region was observed, with the strongest DIF in one item. These results suggest that the current version of I-RODS could be improved or alternative PROMs for patients with GBS could be developed.

**Supplementary Information:**

The online version contains supplementary material available at https://doi.org/10.1007/s00415-026-13684-6.

## Introduction

Guillain–Barré syndrome (GBS) is an immune-mediated polyneuropathy, characterised by a rapidly progressive weakness, sensory deficits, autonomic dysfunction and a typical monophasic disease course [1, 2]. Effective treatments in the acute stage of GBS are intravenous immunoglobulins (IVIg) or plasma exchange (PE), but the clinical response to treatment is highly variable between patients [3]. Patients may show a rapid and full recovery, whilst others show no sign of improvement and require ventilation for months. Most patients require rehabilitation, and a considerable proportion have persistent motor or sensory impairment [4]. In addition to neurological deficits, GBS can exert a profound impact, placing a substantial physical, emotional, and social burden on affected individuals. Residual fatigue and pain are prevalent symptoms of GBS, significantly impacting daily functioning [5]. Severe fatigue in these conditions can substantially limit physical and cognitive abilities and in turn compromising societal participation (e.g. ability to work) and overall quality of life [6, 7] The assessment of GBS during its course is primarily based on clinical outcome measures, including the Medical Research Council Sum Score (MRC sum score) and the GBS Disability Scale (GBS-DS). Patient-Reported Outcome Measures (PROMs) are essential to determine the impact of disease from the patients’ perspective by capturing the outcomes that matter most to patients [8]. Moreover, PROMs are important to monitor disease progression and to evaluate the effect of treatment in clinical practice and trials [9]. The Inflammatory Rasch-built Overall Disability Scale (I-RODS) is one of the first PROMs that is frequently used in clinical practice and in treatment trials in GBS [10, 11]. The I-RODS was designed to assess overall disability (i.e. activity and social participation limitations) and was constructed through a Rasch analytic approach [12]. It was developed in the Netherlands in the Dutch language and consists of items that evaluate aspects of disability, summarised into one total score [13]. Items were developed based on the International Classification of Functioning, Disability and Health (ICF) and other scales to reflect limitation in activity and social participation. However, patients were not directly involved in every stage of the development process. Other potential limitations of the I-RODS are that the scale was initially developed for all types of immune-mediated polyneuropathies and not specifically for GBS and largely in patients from The Netherlands potentially restricting its applicability for international use due to cultural and linguistic variations. This is supported by a previous study, revealing item bias across different regions [11]. To date, I-RODS has not undergone rigorous validation in independent cohorts of patients with GBS. The few studies that have assessed its measurement properties focussed mainly on responsiveness [14] or were based on the same cohort of patients [10, 11, 15].

The aim of the current validation study was to assess structural validity, cross-cultural validity, internal consistency, reliability, construct validity, and floor and ceiling effects of the I-RODS in an international prospective cohort of patients with GBS from various geographic regions.

## Methods

### Study design

This study used data from the International GBS Outcome Study (IGOS), a prospective observational cohort study in which all patients diagnosed with GBS within two weeks of onset of weakness could be included, independent of age, clinical variant, and severity or treatment [16]. Patients were included from the following countries: Argentina, Australia, Bangladesh, Belgium, Canada, China, Denmark, France, Germany, Greece, Italy, Japan, Malaysia, Netherlands, South-Africa, Spain, Switzerland, Taiwan, United Kingdom, and United States of America. Clinical features, diagnostic findings, treatment, disease course, and outcome were collected. The I-RODS was administered at four weeks after inclusion in the cohort, a clinically relevant time point when all patients have reached clinical nadir, which is also frequently used to evaluate primary endpoints in clinical trials [17]. During this period, patients present with considerable heterogeneity in clinical outcomes, which makes this an appropriate time to evaluate disease burden and functional status.

### I-RODS

The I-RODS is a PROM that assesses limitations in activity and social participation in patients with immune-mediated neuropathies. The I-RODS consists of 24 items reflecting limitations in daily activities, with response categories of 0 (“Not possible”), 1 (“Possible with effort”), and 2 (“Easy to perform”). Examples of activities are: “Wash your lower body” and “Turn a key in a lock”. The total score ranges from 0 to 48, with lower scores indicating a higher degree of patient-reported disability [13]. The I-RODS was originally developed in English and officially translated (and back translated) into Chinese, Danish, Dutch, English, French, German, Italian, Japanese, and Spanish. It was administered through paper and pencil by a treating physician. In Bangladesh, no official Bangla translation of the I-RODS was available and due to limited literacy of the patients, physicians verbally administered the questionnaire by providing item-by-item explanations in Bangla and documenting the patients’ responses on their behalf. The I-RODS version used in this study can be found in Supplementary Table 1.

### Statistical analysis

Descriptive statistics of the baseline characteristics of the study population were performed using SPSS (version 28, IBM Corp., Armonk, NY, USA). Definitions of the measurement properties; content validity, structural validity, cross-cultural validity, internal consistency, construct validity, and floor and ceiling effects are presented in Supplementary Table 2. The structural validity of the I-RODS was assessed by Rasch analysis using RUMM2030Plus (RUMM Laboratory, Perth, Australia). Confirmatory Factor Analysis was performed in R, using the lavaan package [18]. A summary of the psychometric properties, analyses and criteria is presented in Table 1.

**Table 1:** Psychometric properties, analyses and criteria

**Measurement property**	**Analysis**	**Description of analysis**	**Indices/indicators**	**Criteria good propert**y
Structural validity Unidimensionality	Series of t–tests (Rasch analysis)	Independent sets of items (8v8 for I-RODS) are used to generate separate person estimates for each individual. These person estimates are then compared to determine whether they are equivalent	Percentage of significant t-tests	Lower 95% binomial CI </= 0.05
	Confirmatory factor analysis (CFA)	Factorial structure examination, if all items load on a single factor (construct) unidimenstionality can be assumed	Chi-square p-value, Scaled Comparative Fit Index (CFI), root mean square error of approximation (RMSEA), Goodness of Fit Index (GFI), Tucker-Lewis Index (TLI) and standardised root mean square residual error (SRMR)	Non-significant chi-square *p*-value CFI > 0.95 RMSEA < 0.06 GFI > 0.95 TLI > 0.95 SRMR < 0.08
Local independence	Rasch analysis	The level of independence of responses to items, such that the measurement is solely focussed on the underlying construct being measured is determined by examining the residual correlation matrix. After controlling for the dominant factor, there should be no important covariance amongst item responses	Residual (Pearson) correlation values (Q3 values)	< 0.20 above the average correlation
Rasch model fit *Overall fit*	Rasch analysis	A summation of the X^2^ values from the individual items. Can be used as a general indication of the magnitude of fit across the item set	X^2^, p-value	> 0.01
*Person fit*		The degree to which a person’s response pattern reflects their overall score	Z-standardised fit residual	± 3
*Item fit*		The degree to which an item works with the rest of the item set to measure an underlying construct. Item anomalies are indicated by the degree of misfit	Z-standardised fit residual; X^2^ p-value	± 2.5; > 0.01
*Response category*		Threshold ordering	Threshold map and category probability curves	All response categories ordered
Internal consistency	Reliability analysis	The inter-relatedness of the items in a scale and, the ability of the measure to reliably order the individuals who are being measured	Person Separation Index (PSI) (Rasch) Cronbach’s alpha (α)	> 0.85 = excellent > 0.70
Cross-cultural validity	Differential item Functioning (DIF) analysis with Analysis of Variance (ANOVA)	People from different groups with the same level of the construct should have the same probability of giving a certain response to an item. If these probabilities are not the same, there is DIF	*p*-value	> 0.05
Construct validity	Correlation analysis	Examining the relationship between the I-RODS scores and a clinical outcome measure	Spearman correlation coefficient (R)	> 0.50
Floor and ceiling effects	Descriptive statistical analyses	Percentages of patients with the lowest and highest possible scores indicate ineffective differentiation amongst individuals with different construct levels	Percentages (%)	< 15%
Targeting	Rasch analysis	Comparing the ‘ability’ distribution of individuals with the ‘difficulty’ distribution of the items to see if the instrument aligns with the population estimates of the underlying construct being measured	Person-item threshold distribution	Bell curve

### Structural validity

#### Confirmatory factor analysis

Prior to applying Rasch analysis to assess the fit of the model, we examined whether the data met the core assumption of unidimensionality, which implies that scores on each item can be summed into one total score to determine the impact of GBS amongst patients. We hypothesised that a one-factor confirmatory factor analysis will reveal that all items measure the same underlying construct, i.e., the outcome that the PROM intends to measure (in this case limitations in activity and social participation). It is assessed through multiple items that represent the outcome [19]. This implies that scores on each item can be summed into one total score to determine the impact of GBS amongst patients.

### Rasch analysis

The I-RODS was developed using Rasch methods, and therefore we evaluated whether the data fits the Rasch model in an independent validation sample of patients with GBS enrolled in IGOS, including patients from diverse geographic regions. To examine whether all items were contributing to the underlying construct of disability, all items were assessed for individual fit to the Rasch model (non-significant at Bonferroni-adjusted chi-square p-value, standardised (z-score) fit-residuals within ± 2.5). Additionally, the overall chi-square p-value is reported for the scale, which provides an indication of scale fit. This reflects how well the items in the PROM effectively measure the same underlying construct, and whether the responses are consistent with the expectations of the model [20]. Response category functioning was assessed to ensure that item response categories were operating as intended. The locations of the crossover points between adjacent response categories (the thresholds) should logically increase across the underlying trait, in order to represent distinctly separate response categories. It is acknowledged that Rasch fit statistics within RUMM2030 can become overpowered by large sample sizes [21]. Therefore, for the tests of item fit, scale fit, differential item functioning and unidimensionality, a random sample of n = 600 was taken in order to aid interpretation and limit type II error. Although a minimum of 500 participants is suggested, we opted for a slightly larger sample to account for the presence of extreme values (i.e. participants scoring at the floor or ceiling of the scale). The complete sample was used for all other fit indices.

#### Unidimensionality

When generating a total score from a set of items, it is necessary that all items measure the same underlying construct. This scale characteristic is known as unidimensionality and this is an assumption of the Rasch model. In order to test unidimensionality within the Rasch analysis, the item loadings of a residual principal components analysis are used to identify the two item sets within a scale that diverge the most. These two independent sets of items are then used to derive two separate person estimates, which should be equivalent if the notion of unidimensionality holds. A series of paired t-tests is then used to compare the separate person estimates for each individual and unidimensionality is evident when the person estimates do not differ in more than 5% of cases, with a lower bound 95% confidence interval applied [20, 22].

#### Local independence

Local independence means that responses to items are not correlated beyond what is explained by the underlying construct that is being measured, ensuring that each item provides independent information. All items were tested for local dependency, where the residual correlation Q3 criterion cut point to indicate dependency was taken as 0.2 above the average residual correlation [23].

### Cross cultural validity

Differential Item Functioning (DIF) is a form of measurement bias which occurs when an item operates differently between pre-defined groups of individuals, conditional on the level of the construct being held steady. DIF was assessed for sex (male/female), age group (0–52/53 +), defined by the median value and, DIF by geographic region (Bangladesh/Northern-Europe/Southern-Europe/North America/Asia/Other) providing a measure of cross-cultural validity.

### Internal consistency

Internal consistency refers to the interrelatedness amongst items within an instrument and when it is sufficiently high, it indicates that all the items of the instrument are measuring the same underlying construct consistently. Internal consistency reliability indices were taken as the person separation index (PSI) and Cronbach’s alpha.

### Construct validity

We evaluated construct validity by comparing the I-RODS scores to frequently used clinical outcome measures for GBS, i.e. the GBS-DS [24, 25], MRC sum score [26], Overall Neuropathy Limitations Scale (ONLS) [27], and a PROM EQ-5D-5L [28]. We hypothesise a moderate to strong negative correlation between the I-RODS and the GBS-DS with Spearman’s correlation coefficient (*ρ*) expected to be lower than −0.6. We expect a moderate positive correlation between the I-RODS and the MRC sum score with Spearman's correlation coefficient anticipated to be greater than 0.5. Furthermore, we anticipate a strong negative correlation between the I-RODS and the ONLS with Spearman's correlation coefficient expected to be lower than −0.7. For the EQ-5D-5L domains mobility and self-care we expect a moderate to strong negative correlation with the I-RODS, with a Spearman's correlation coefficient of lower than −0.6. For the domain usual activities, we expect a strong negative correlation with a Spearman's correlation coefficient of lower than −0.7.

### Floor and ceiling effects

Percentages of patients with the lowest and highest possible scores were calculated to check for potential floor or ceiling effects. The recommended limit at either end is 15% [29]. The presence of these effects suggests that the PROM may be limited in differentiating between individuals with different levels of the construct. This leads to limitations in detecting improvements and deterioration within individual patients. Floor and ceiling effects indicate a compromised measurement range, where the measurement range of the instrument does not cover the relative location range of the target population. Scale targeting was also assessed visually to provide an indication of the relative distribution of item and person locations.

## Results

### Study population

**Fig. 1:**
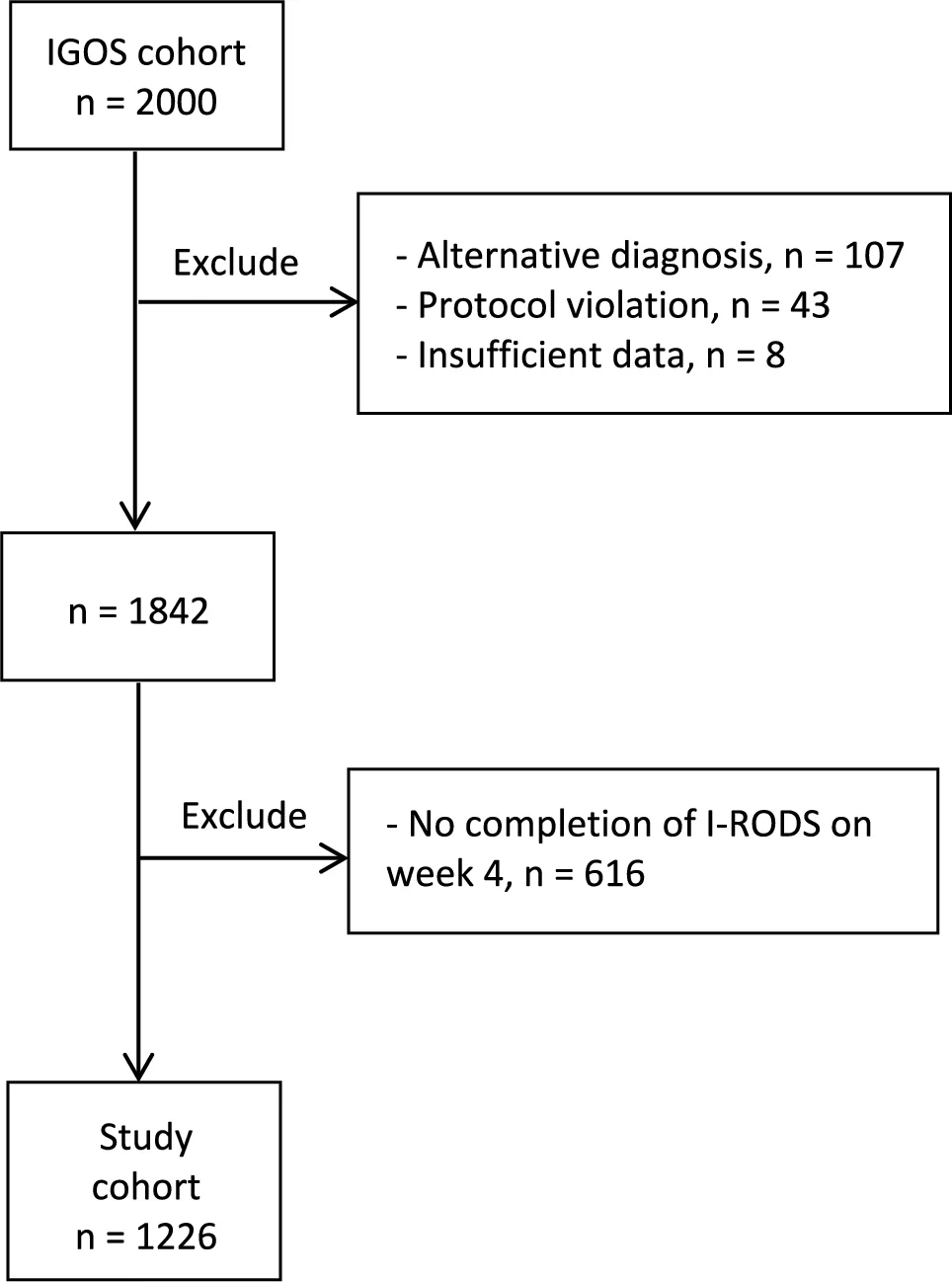
Flow diagram study population

From the IGOS 2000 cohort we excluded 107 (5%) patients with an alternative diagnosis, 43 (2%) patients with a protocol violation, 8 (0.4%) patients with insufficient data, and 616 (31%) patients who did not complete the I-RODS at week 4. These excluded patients without I-RODS did not differ from the included patients with respect to demographical (age: p = 0.72 and sex: p = 0.59) and clinical characteristics, with the exception of the GBS-DS (p < 0.001), which was slightly lower (corresponding with slightly less severe disability) in included patients (median 2; IQR 1–4) than in excluded patients (median 3; IQR 1–4).The remaining group of 1226 patients were eligible for the current study (Fig. 1). The median age was 51 years (IQR 35–64 years), with a male to female ratio of 1.5. The median I-RODS score was 28 (IQR 10–41) at week 4. Sample demographics are presented in Table 2.

**Table 2:** Patient characteristics

I-RODS	Overall	Bangladesh	Asia^1^	Northern^2^ Europe	Southern^3^ Europe	North^4^ America	Other^5^
Demographics							
N	1226	189	189	398	260	125	65
Age in years, median (IQR)	51 (35–64)	30 (18–45)	52 (39.5–64.5)	56 (41–67)	56 (41–68)	53 (34.25–63)	46 (30.5–60.5)
Sex (%)							
Male	64.0	64.0	57.7	58.8	60.0	56.8	64.6
Female	36.0	36.0	42.3	41.2	40.0	43.2	35.4
Clinical feature							
GBS disability score (%)							
0	5.0	0.5	11.1	3.5	5.8	5.6	4.6
1	22.3	9.5	34.9	15.1	26.9	30.4	32.3
2	29.4	27.0	29.6	33.2	30.4	21.6	24.6
3	16.2	18.0	6.9	19.8	13.5	20.8	16.9
4	21.9	34.4	14.3	21.6	21.5	16.8	20.0
5	5.1	10.6	3.2	6.0	1.9	4.8	1.5
MRC sum score, median (IQR)	55 (42–60)	36 (22–48)	59 (48–60)	56 (47–60)	58 (48–60)	59 (52–60)	54 (47–60)
ONLS, mean (SD)	4.7 (3.7)	6.9 (3.7)	3.4 (3.5)	5.0 (3.4)	4.0 (3.5)	4.3 (3.6)	4.0 (3.4)
EQ-5D-5L, median (IQR)							
Mobility	2 (0–4)	3 (1–4)	1 (0–2)	2 (1–4)	1 (0–3)	1 (0–3)	1 (0–3)
Selfcare	1 (0–3)	3 (1–4)	0 (0–2)	1 (0–2)	1 (0–2)	0 (0–2)	0 (0–2)
Usual activities	2 (1–4)	3 (1–4)	1 (0–2)	3 (1–4)	1 (0–3)	2 (1–3)	0 (1–3)
GBS forms and variants (%)							
Motorsensory form	57.7	21.2	45.0	70.1	67.7	70.4	60.0
Motor form	24.7	76.7	27.5	11.3	15.4	5.6	21.5
MFS and MFS- GBS overlap	12.2	1.6	20.6	13.1	10.0	16.8	13.8
Other^6^	3.4	0.5	6.4	3.1	5.5	4.8	3.0
Treatment (%)							
No treatment	19.0	74.6	6.3	12.6	4.2	7.2	15.4
IVIg	67.2	7.9	58.2	83.7	80.0	84.8	80.0
Plasma exchange	12.5	11.6	35.4	3.0	15.8	8.0	1.5
Other	1.3	5.8	–	0.8	–	–	3.1

*I-RODS* Inflammatory Rasch overall disability scale, *IQR* Inter quartile range, *MFS* Miller fisher syndrome, *IVIg* Immunoglobulins, *ONLS* Overall neuropathy limitations scale, *MRC* Sum score medical research counsil sum score, *EQ-5D-5L* EuroQol 5 dimensions 5 levels

^1^Asia: Japan, Malaysia, Taiwan and China (Bangladesh excluded because of low socio-economic status)

Northern Europe^2^: Belgium, Denmark, Germany, Netherlands, United Kingdom

Southern Europe^3^: France, Greece, Italy, Spain, Switzerland

North America^4^: Canada and the United States of America

^5^Other countries: Argentina, Australia and South Africa

^6^Other clinical variants: pharyngo-cervical-brachial, pure sensory, ataxic or other variant

### Structural validity

#### Confirmatory factor analysis

The fit of a single-factor model showed a chi-square value of 1049 (df 252), p < 0.001. The RMSEA = 0.069 (95% CI = 0.065–0.073); CFI = 0.998; TLI = 0.997; SRMR = 0.038; GFI = 0.999 (Table 1). These results are somewhat contradictory, as the chi-square result suggests a significant misfit from the single-factor model, but all of the other confirmatory factor analysis indices suggest a good fit to the single-factor model. The hypothesis that all items would fit a single-factor model can therefore not be fully accepted or rejected. However, the chi-square fit statistic may be sensitive to the large sample size, and the supplementary fit indices indicate that the items could be taken forward into a Rasch analysis.

### Rasch analysis

**Fig. 2:**
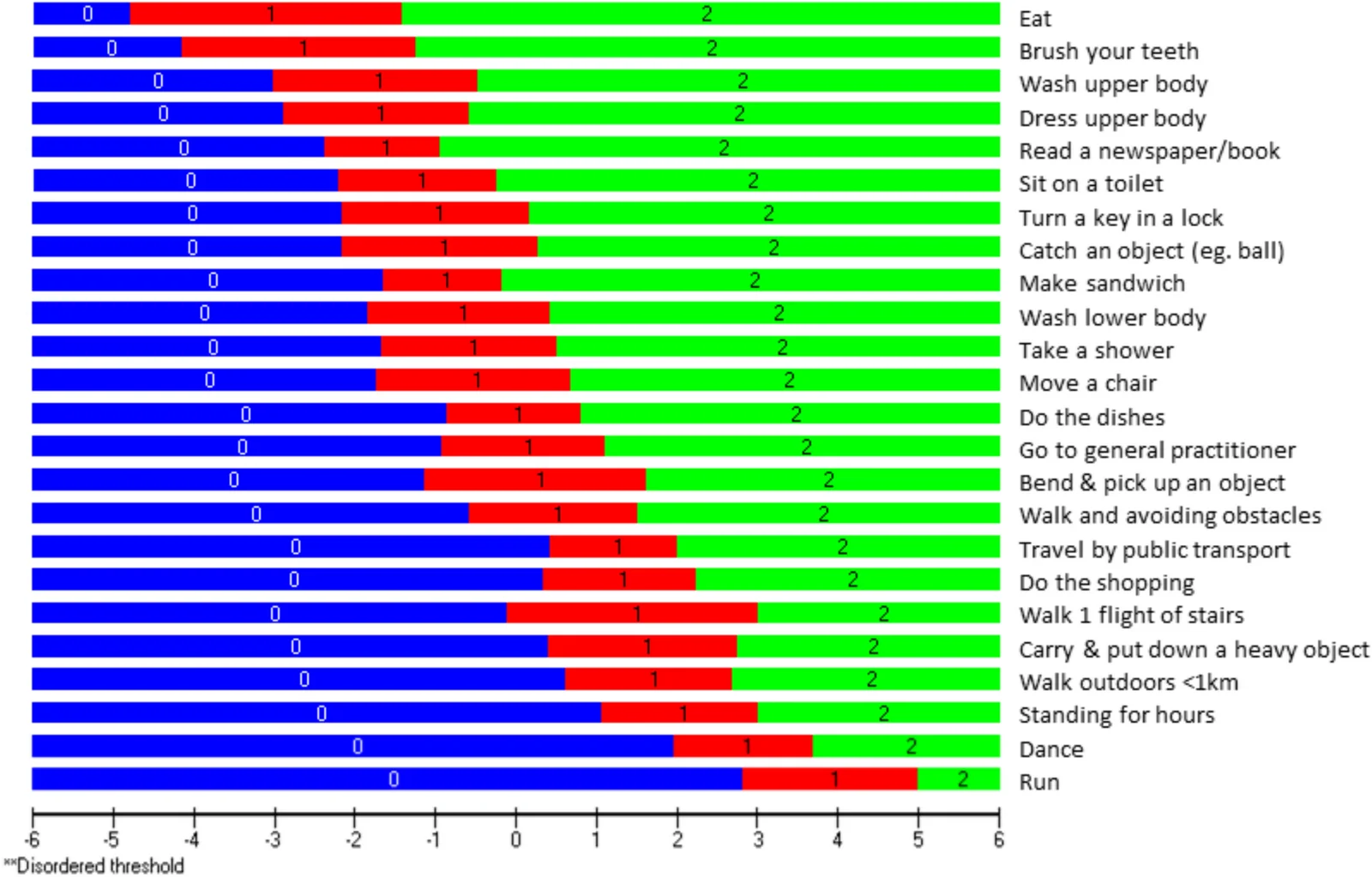
Threshold map for I-RODS in IGOS study cohort

A summary of the overall fit indices is provided for both the full sample and the n = 600 subsample (Table 3). Additionally, a complete table of the individual item fit is provided in Table 4 and Supplementary Table 3, and pairwise local dependency is provided in Supplementary Tables 4 and 5. The overall fit of the responses on the I-RODS deviates significantly from the Rasch model based on the chi-square fit statistic, reflecting a poor overall fit. A good person fit was observed, with fewer than 1% (8/1224) of the individuals falling outside of the accepted fit residual range. Misfit anomalies were observed for 6 items: “Are you able to catch an object (e.g. a ball)”, “Are you able to wash your lower body”, “Are you able to take a shower”, “Are you able to brush your teeth” and “Are you able to read a book/newspaper”. The items “Are you able to read a book/newspaper” and “Are you able to catch an object (e.g. a ball)” demonstrated the largest under-discrimination anomalies, indicating that these items failed to accurately discriminate between different disability levels. This suggests that these two items potentially measure a different construct compared to the other items within the scale. All response categories operated as intended, with thresholds for all items demonstrating appropriate threshold ordering (Fig. 2).

**Table 3:** Fit of the Rasch model

	Full sample	Subsample	Target values
total n- (extremes) = valid n	1226–(248) = 978	600–(116) = 484	-
Number of items	24	24	-
Overall scale fit (Chi-square p)	*p < 0.001	*p < 0.001	*p* > 0.01
Individual item fit residuals	6/24 misfitting	6/24 misfitting	within ± 2.5
Individual item fit (Chi-square p)	7/24 misfitting	2/24 misfitting	*p* > 0.05 (Bonferroni adj)
Person Fit Residuals	< 1% outside range	< 1% outside range	within ± 3.0
Unidimensionality	12.13% (CI 10.7–13.5%)	14.46% (CI 12.5–16.4%)	% of significant t-tests < 5%
Local dependency	15/276 pairwise significant	20/276 pairwise significant	< 0.2 above average Q3
Targeting	Satisfactory	Satisfactory	aligned
% sample at floor (min score)	10.1%	8.3%	-
% sample at ceiling (max score)	9.4%	11.0%	-
% sample at floor and ceiling (combined)	19.5%	19.3%	< 15%
Person separation index (PSI)	0.95	0.95	> 0.85
Response category threshold ordering	All ordered	All ordered	All ordered
DIF-by-sex	No DIF	No DIF	*p* > 0.05 (Bonferroni adj)
DIF-by-age group	1/24 Uniform; 2/24 Non-uniform	No DIF	*p* > 0.05 (Bonferroni adj)
DIF-by-ethnicity	15/24 Uniform; 5/24 Non-uniform	11/24 Uniform; 3/24 Non-uniform	*p* > 0.05 (Bonferroni adj)

*DIF* Differental item functioning

^*^Significant values *p *< 0.05

**Table 4:** Individual item fit for full sample (*n* = 1226)

Item	Statement	Location	SE	Fit residual	Probability
1	Bend forward and pick something up	0.256	0.069	− 2.236	0.076395
2	Remain standing for a long time period, e.g. several hours	2.033	0.069	0.64	0.027907
3	Walk up a flight of stairs	1.450	0.07	− 2.235	0.005942
4	Run	3.907	0.082	− 1.169	0.316908
5	Walk outdoors, up to max 1 kilometre	1.665	0.069	− 1.359	0.081432
6	Walk while avoiding obstacles	0.465	0.068	− 2.186	0.233463
7	Dance	2.828	0.084	− 1.39	0.396509
8	Travel by public transport	1.213	0.073	− 1.717	0.000581^+^
9	Turn a key in a lock	− 0.993	0.073	2.027	0.003296
10	Carry and put down a heavy object	1.589	0.07	− 0.814	0.654511
11	Move a chair	− 0.524	0.071	− 2.11	0.005912
**12**	**Catch an object (e.g. a ball)**	− **0.936**	**0.074**	*4.483	**0**^**+**^
13	Wash your upper body	− 1.746	0.077	*2.739	0.010976
14	Wash your lower body	− 0.712	0.071	*3.519	0.007661
15	Take a shower	− 0.574	0.07	*4.697	0.000156^+^
16	Brush your teeth	− 2.693	0.085	2.257	0.001171^+^
17	Sit on/go to a toilet	− 1.208	0.073	− 0.13	0.058559
18	Dress your upper body	− 1.736	0.076	− 1.905	0.003777
19	Eat	− 3.089	0.089	1.373	0.777803
20	Do the dishes	− 0.015	0.079	− 1.964	0.000052 +
21	Make a sandwich	− 0.914	0.082	− 0.035	0.043545
22	Do the shopping	1.292	0.073	*4.276	0.000065^+^
23	Go to the general practitioner	0.102	0.073	− 1.1	0.002904
**24**	**Read a book/newspaper**	− **1.658**	**0.076**	*8.915	**0**^**+**^

Fit residuals within ±2.5 indicate acceptable fit

*SE* Standard error

Misfit based on fit residuals are marked (*), misfit based on chi-square (p <.05) are marked (^+^), and the item with the largest misfit is shown in bold

#### Unidimensionality

The series of t-tests demonstrated that 12.1% (CI 10.7–13.5%) of the full sample and 14.5% (CI 12.5–16.4%) of the subsample report significantly different person estimates. This is more than the 5% that would be expected naturally and is suggestive that the I-RODS is not a unidimensional measurement instrument measuring solely limitations in activity and social participation.

#### Local independence

Lack of local independency was identified within the item set, with 15 of the 276 (5.4%) pairwise correlations suggesting dependencies. The largest of these dependencies was found between item “Are you able to wash your upper body” and “Are you able to wash your lower body”.

### Cross cultural validity

There was no substantive DIF observed for sex or age group. However, significant DIF was evident for geographic region, with 11 of the 24 items displaying systematic uniform differences in item functioning. A DIF summary table is provided in Supplementary Table 6, along with the DIF-by-geographic region plots for the individual items in Supplementary Figs. 1– 24. The item “Are you able to remain standing for a long time period” was the item displaying the largest degree of DIF by geographic region, and this is presented in Fig. 3, where it can be seen that this is considered to be an ‘easier’ item in Asia than it is in Northern Europe. In order to provide a comparison between the different geographical regions, the overall expected values for each geographical region are presented for each item in Supplementary Table 7.

**Fig. 3:**
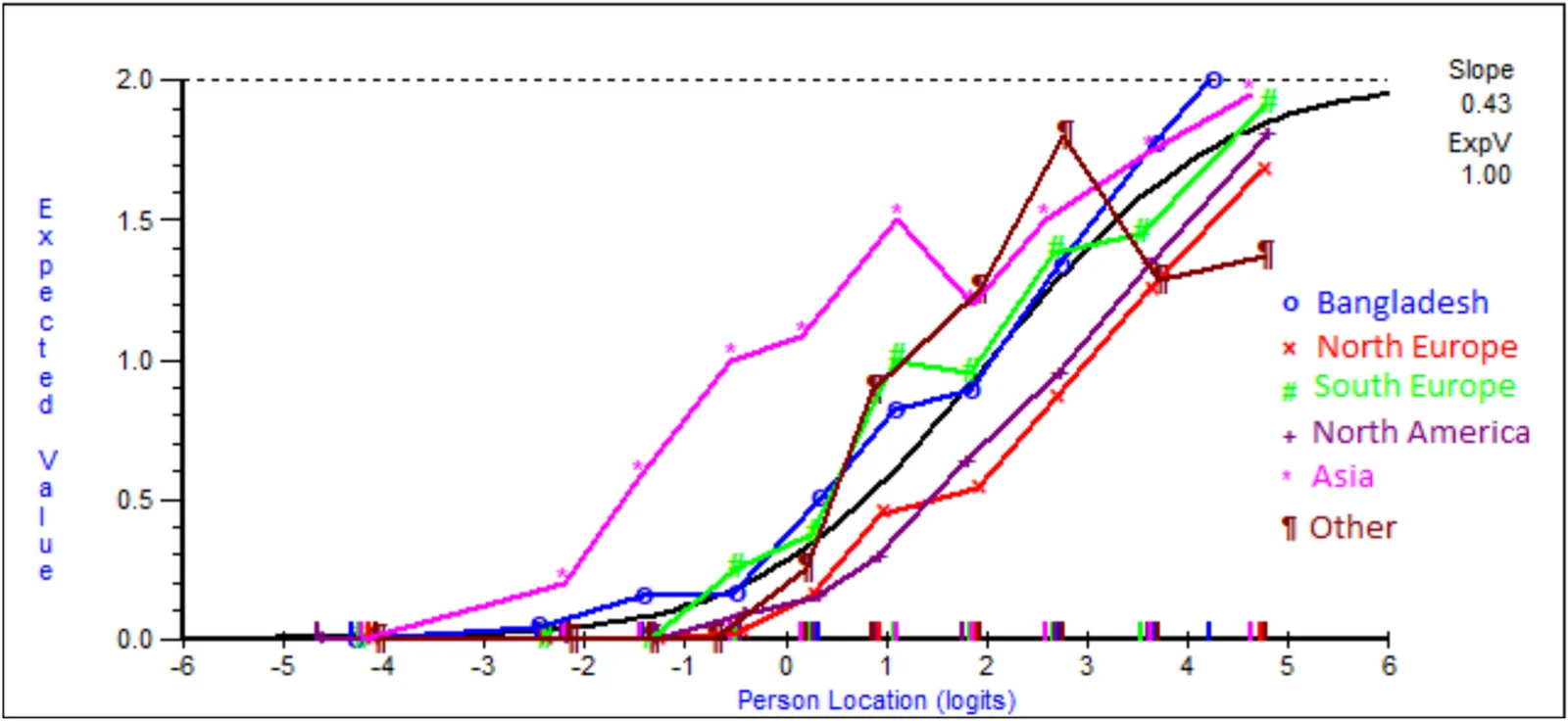
DIF by region for item 2 “Remain standing for a long time period”. *Person location refers to the level of disability of each patient with mean=0 and SD=1 and the expected value refers to the expected item response based on the level of disability

### Internal consistency

The I-RODS demonstrated sufficient internal consistency, with a Person Separation Index (PSI) of 0.95 with extremes and 0.96 without extremes and a Cronbach’s alpha of 0.98.

### Construct validity

The expected and observed correlations between the I-RODS and other measures are presented in Table 5. As anticipated, a strong negative correlation was observed between the I-RODS and GBS-DS, indicating that lower scores on the I-RODS are associated with higher scores on the GBS-DS. A strong positive correlation was found between the MRC sum score and the I-RODS, with higher scores on the I-RODS associated with higher scores on the MRC sum score. A strong negative correlation was observed between the I-RODS and the ONLS, where higher scores on the I-RODS scores were associated with lower scores on the ONLS. A strong negative correlation was also found between the I-RODS and the three EQ-5D dimensions. Higher scores on the I-RODS were linked to lower scores on the mobility, usual activities, and self-care dimensions of the EQ-5D.

**Table 5:** Expected and observed correlations

Outcome measure	I-RODS	
	Expected correlation	Observed correlation
GBS-DS	≤ −0.60	− 0.91
MRC sum score	≥ 0.50	0.77
ONLS	≤ −0.70	− 0.90
EQ-5D-5L Mobility	≤ −0.60	− 0.86
EQ-5D-5L Self care	≤ −0.60	− 0.86
EQ-5D-5L Usual activities	≤ −0.70	− 0.81

*I-RODS* Inflammatory Rasch-built overall disability scale, *GBS-DS* GBS disability scale, *ONLS* Overall neuropathy limitations scale, *MRC sum score* Medical research counsil sum score, *EQ-5D-5L* EuroQol 5 dimensions 5 levels

### Floor and ceiling effects

**Fig. 4:**
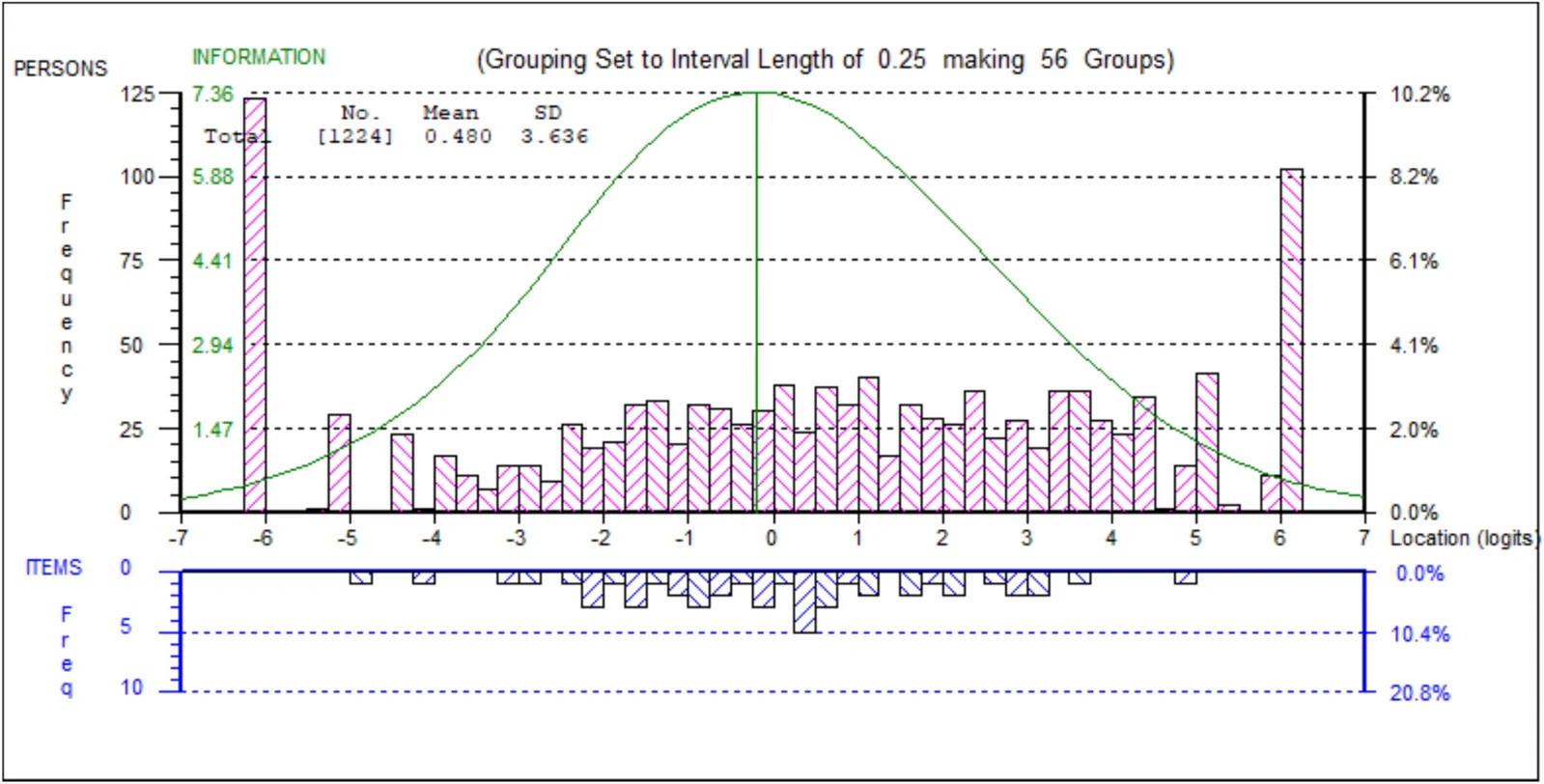
Person-item threshold distribution. *Person-Item Threshold Distribution depicts the distribution of item location (disability levels of the patients) relative to the distribution of item location (the disability levels represented by the scale of the items. The x-axis represents the continuum of disability with a frequency distribution of the GBS patient locations plotted above the x-axis and a frequency distribution of the I-RODS item locations plotted below the x-axis

The I-RODS revealed that 10.1% of the patients obtained the lowest possible score and 9.4% of the patients obtained the highest possible score (Fig. 4). This is reflected in the distribution of patients that is somewhat spread out and evenly distributed, whereas we would expect a normal distribution. The person-item threshold distribution is presented in Fig. 4.

## Discussion

This study evaluated the measurement properties of the I-RODS, a widely utilised PROM for patients with GBS in both clinical practice and research settings. Using Rasch methodology, the analysis of an extensive international patient cohort revealed adequate internal consistency, acceptable targeting, and good construct validity. However, there are several notable limitations in the current version of the I-RODS.

First, I-RODS demonstrated a lack of unidimensionality. This suggests that multiple constructs instead of a single construct were measured by I-RODS and that the current practice of summing up scores on the items into one total score is inappropriate. The apparent lack of unidimensionality could also be caused by the high degree of local dependency that was found amongst the I-RODS items, which was observed across multiple pairwise correlations. This high level of local dependency could also be artificially inflating the high internal consistency reliability values (PSI = 0.95; Cronbach’s alpha = 0.98). In addition, these findings to some extent also could explain the inconclusive confirmatory factor analysis results, where the single-factor model was rejected by the Chi-square test, but all of the supplementary fit indices indicated a good fit. Taken together, these results indicate that the assumed unidimensional structure does not hold and that scores should be interpreted with caution, as they may not accurately reflect limitations in daily activity and social participation.

Second, individual item misfit was present in six items, with two items (“Are you able to read a book/newspaper” and “Are you able to catch an object (e.g. a ball)” displaying problematic under-discrimination. These two items should be reviewed by experts based on their content for revision or possible removal, as they may not be appropriate for GBS.

Third, although there was no apparent floor and ceiling effect, as the percentage at each end falls below 15% and the targeting of the I-RODS to patients with GBS was considered acceptable, there was a combined total of 19.5% of patients scoring at the floor or ceiling. This reflects a limitation of the scale because it fails to capture 19.5% of the GBS population (within our sample), which is approximately one in five patients. This tendency towards the floor and ceiling could be the result of the absence of content validity of the I-RODS or that the I-RODS does not fully capture the disability range found within GBS patients [30]. Reviewing the relevance, comprehensiveness, and comprehensibility of each item by patients with GBS and the inclusion of easier as well as more challenging items could improve the targeting of the questionnaire and minimise the proportion at the floor and ceiling. Lastly, DIF was detected for geographical region but not for age and sex. Patients from different regions vary significantly in the way that items operate, probably attributable to cultural differences. For example, the largest DIF was found for the item “Remain standing for a long time period”, with the biggest difference between regions noted between Asia and Northern Europe. This could be due to the interpretation of what is considered to be a ‘long time,’ or it could reflect the relative difference in the amount of time spent standing in the different regions. That the Asian region considered this item to be ‘easier’ suggests that standing for a long period is easier for respondents from Asia than it is for respondents from Northern Europe. The presence of DIF for region can limit the comparability of scores across different languages or regions, which can be problematic given that the I-RODS is used globally. Our findings regarding the cross-cultural validity of the I-RODS are in line with previous research [11]. However, the current study extends on previous analyses by including patients from Asia and a larger number of patients from other geographic regions.

The existing literature on the validation of the I-RODS is very limited. Only a few studies have assessed its responsiveness and construct validity, with the quality of the methods of the studies on responsiveness being suboptimal; for example, the small sample of 55 patients was insufficient to adequately assess responsiveness [10, 14, 15]. For the construct validity of the I-RODS, the predefined hypotheses on the correlation between the I-RODS and GBS-DS, MRC sum score, ONLS, and EQ-5D-5L mobility, usual activities, and self-care were confirmed with strong correlations (−0.91–0.77). Construct validity was assessed in previous studies by examining the correlations between the I-RODS and the EQ-5D and a performance-based test (vigori-dynamometer) [10, 15]. However, no predefined hypotheses were formulated, which is currently a requirement for the evaluation of construct validity according to the COSMIN guideline [30]. In a recent systematic review on the measurement properties of PROMs for patients with polyneuropathies, we formulated hypotheses for testing the construct validity of the I-RODS and other PROMs, and the results of the studies mentioned above were not in line with these hypotheses [31]. Reasons for this inconsistency are unclear. Moreover, the involvement of patients in the development of the I-RODS was indirect, as they reviewed items for another questionnaire in a prior study, from which some items were later incorporated into the I-RODS [32]. Patients completed the preliminary version of the I-RODS. However, they were not involved in a concept elicitation phase, which is considered a crucial step for establishing content validity according to the COSMIN guideline [19, 33]. The importance of patient involvement and qualitative studies in establishing adequate content validity is also emphasised by the US Food and Drug Administration (FDA) [9]. This scarcity and methodological limitations of research on the measurement properties of the I-RODS underscore the importance of the current findings and the implications for the use of the current I-RODS scale in future clinical trials within a GBS sample.

The differences in findings between this study and the development study may be attributed to several factors. First, this study included a larger, multinational sample consisting solely of GBS patients, whereas the development study involved a relatively small Dutch cohort with a mixed patient population (GBS, chronic inflammatory demyelinating polyneuropathy, and gammopathy-related polyneuropathy). Second, the development study assessed clinically stable patients and in a later stage of the disease, whilst this study used data collected at the four-week timepoint, when the condition of patients was likely to be more severe. Third, the advancement of analytical methodology, such as for example, the assessment of local dependency, which is now conducted with greater rigour than at the time the I-RODS was developed.

Considering the evaluation of the measurement properties of the I-RODS, our results suggest a need for a more psychometrically valid PROM in the context of assessing disability in patients with GBS. A possible alternative could be the use of high-quality generic PROMs from the Patient-Reported Outcomes Measurement Information System (PROMIS) [34]. We recommend using multiple domains of PROMIS that are considered to be relevant for patients with GBS, such as fatigue, physical function, and ability to participate in social roles and activities.

The high-quality nature of the data, achieved through a predefined study protocol, prospective collection, and regular data quality controls during the study conduct, is a key strength of this validation study. The international cohort, including patients from both high- and low-income countries, contributed to the robustness of the study and allowed for the analysis of cross-cultural validity. Furthermore, the substantial sample size provided an adequate reflection of the GBS population. The availability of multiple outcome measures in IGOS allowed us to evaluate construct validity by comparisons with various other relevant outcome measures. A limitation of the study is the focus on a single time point rather than multiple time points. Test–retest reliability and responsiveness were beyond the scope of this study. Future studies should consider adopting a longitudinal approach with high-quality methods. Another limitation is that, in Bangladesh, the questionnaires were administered verbally by the physicians, which may have introduced interviewer bias and differences in interpretation of the items. Nonetheless, this approach addresses literacy-related barriers and facilitates broader participation and representativeness of the study population. A final limitation is the relative underrepresentation of the most severely affected patients within this cohort. This can be reasonably explained by the difficulty these patients experience in completing the I-RODS in the acute phase of GBS. However, it is important to note that the primary aim of this study is to assess the measurement properties of the PROM, rather than to provide a representative depiction of GBS disease burden. In conclusion, this study found evidence for adequate internal consistency, acceptable targeting, and good construct validity but insufficient structural validity and cross-cultural validity. This means that the scores on the I-RODS may not accurately reflect one construct, namely, limitations in daily activities and social participation. Some items seem closely related to each other in a way that suggests the PROM contains a degree of dependency or redundancy. Additionally, some items might be picking up on another construct beyond limitations in daily activity and social participation. Moreover, it might not perform consistently across different cultures or languages, and the difficulty of the items doesn’t always match the level of disability observed in this patient sample. We therefore propose to revise and improve the current version of the I-RODS, use another PROM, or develop a new PROM focussed on various relevant domains of quality of life relevant for GBS patients.

## Supplementary Information

Below is the link to the electronic supplementary material.Supplementary file1 (DOCX 364 KB)

## Data Availability

In compliance with the General Data Protection Regulation, patient data cannot be disclosed as patient approval has not been obtained for sharing coded data. Syntax and statistical outputs are available from the corresponding author upon reasonable request.
