# Supplementary material for: Measurement properties of the Inflammatory Rasch-built Overall Disability Scale (I-RODS) in patients with Guillain–Barré syndrome
Source: J Neurol. 2026 Feb 25;273(2):164. doi: 10.1007/s00415-026-13684-6 (PMC12935710; doi:10.1007/s00415-026-13684-6)
Supplement: Supplementary file 1 — Supplementary file1 (DOCX 367 KB) [file 415_2026_13684_MOESM1_ESM.docx]

**Supplementary Tables and Figures**

**Supplementary Table 1: Inflammatory Rasch-built Overall Disability Scale (I-RODS)**

|  | **Activities** | **Difficulty performing this activity** | | | |
| --- | --- | --- | --- | --- | --- |
|  |  | **0** | **1** | **2** |  |
|  | **Are you able to:** | **Not possible** | **Possible with effort** | **Easy to perform** | **Not applicable** |
| 1 | Bend forward and pick something up |  |  |  |  |
| 2 | Remain standing for a long time period, e.g. several hours |  |  |  |  |
| 3 | Walk up a flight of stairs |  |  |  |  |
| 4 | Run |  |  |  |  |
| 5 | Walk outdoors, up to max 1 kilometer |  |  |  |  |
| 6 | Walk while avoiding obstacles |  |  |  |  |
| 7 | Dance |  |  |  |  |
| 8 | Travel by public transport |  |  |  |  |
| 9 | Turn a key in a lock |  |  |  |  |
| 10 | Carry and put down a heavy object |  |  |  |  |
| 11 | Move a chair |  |  |  |  |
| 12 | Catch an object (e.g. a ball) |  |  |  |  |
| 13 | Wash your upper body |  |  |  |  |
| 14 | Wash your lower body |  |  |  |  |
| 15 | Take a shower |  |  |  |  |
| 16 | Brush your teeth |  |  |  |  |
| 17 | Sit on/ go to a toilet |  |  |  |  |
| 18 | Dress your upper body |  |  |  |  |
| 19 | Eat |  |  |  |  |
| 20 | Do the dishes |  |  |  |  |
| 21 | Make a sandwich |  |  |  |  |
| 22 | Do the shopping |  |  |  |  |
| 23 | Go to the general practitioner |  |  |  |  |
| 24 | Read a book/ newspaper |  |  |  |  |

This version was administered to patients as part of the IGOS study.

**Supplementary Table 2: Definitions of the measurement properties according to COSMIN [33].**

| Measurement property | Definition of the measurement properties | |
| --- | --- | --- |
| **Content validity** | The degree to which the content of a PROM is an adequate reflection of the construct to be measured. It refers to the relevance, comprehensiveness, and comprehensibility of the instrument | |
| **Structural validity** | | The degree to which the scores of an instrument are adequate  reflections of the dimensionality of the construct to be measured |
| **Internal consistency** | | The degree of the interrelatedness among the items |
| **Cross-cultural validity** | | The degree to which the performance of the items on a translated or  culturally adapted instrument is an adequate reflection of the  performance of the items of the original version of the instrument |
| **Construct validity** | | The degree to which the scores of an instrument are consistent with hypotheses (for instance with regard to internal relationships, relationships to scores of other instruments, or differences between relevant groups) based on the assumption that the instrument validly measures the construct to be measured |
| Floor and ceiling effects | | The percentage of patients with the highest or lowest possible score, respectively |

Abbreviations**:** COSMIN = COnsensus-based Standards for the selection of health Measurement INstruments

**Supplementary Table 3: Individual item fit for subsample (n=600)**

| Item | Statement | Location | SE | FitResid | Prob |
| --- | --- | --- | --- | --- | --- |
| I0001 | RODS_bend_pick_up | 0.121 | 0.096 | -1.837 | 0.338557 |
| I0002 | RODS_standing_long | 1.837 | 0.097 | 0.155 | 0.075961 |
| I0003 | RODS_walk_up_stairs | 1.33 | 0.097 | -1.963 | 0.005701 |
| I0004 | RODS_run | 3.594 | 0.112 | -0.895 | 0.405165 |
| I0005 | RODS_outdoors_max_1km | 1.552 | 0.096 | -0.445 | 0.347771 |
| I0006 | RODS_walk_avoid_obstacles | 0.399 | 0.094 | -2.072 | 0.48974 |
| I0007 | RODS_dance | 2.802 | 0.121 | -1.018 | 0.593175 |
| I0008 | RODS_public_transport | 1.117 | 0.101 | -0.964 | 0.044096 |
| I0009 | RODS_turn_key_in_lock | -0.795 | 0.099 | 1.972 | 0.027319 |
| I0010 | RODS_carry_put_down_heavy_object | 1.476 | 0.096 | -0.76 | 0.957435 |
| I0011 | RODS_move_chair | -0.482 | 0.098 | -1.61 | 0.354452 |
| I0012 | RODS_catch_object | -0.911 | 0.101 | 3.204 | 0.000217 |
| I0013 | RODS_wash_upper_body | -1.563 | 0.105 | -1.714 | 0.677723 |
| I0014 | RODS_wash_lower_body | -0.646 | 0.098 | -2.917 | 0.092387 |
| I0015 | RODS_shower | -0.487 | 0.097 | -3.19 | 0.020668 |
| I0016 | RODS_brush_teeth | -2.419 | 0.115 | 2.679 | 0.002376 |
| I0017 | RODS_toilet | -1.234 | 0.101 | -0.605 | 0.139434 |
| I0018 | RODS_dress_upper_body | -1.523 | 0.104 | -1.217 | 0.01321 |
| I0019 | RODS_eat | -3.063 | 0.124 | 1.484 | 0.132499 |
| I0020 | RODS_dishes | 0.072 | 0.109 | -0.154 | 0.004714 |
| I0021 | RODS_sandwich | -0.779 | 0.111 | 0.691 | 0.266525 |
| I0022 | RODS_shopping | 1.136 | 0.102 | -3.006 | 0.017181 |
| I0023 | RODS_general_practitioner | 0.049 | 0.102 | -2.075 | 0.179399 |
| I0024 | RODS_read | -1.586 | 0.104 | 6.461 | 0 |
|  |  | **Bonf** | **0.05** | **24** | **0.002083** |

**Supplementary Table 4: local dependency subsample (n=600)**

| Statement | Item | I0001 | I0002 | I0003 | I0004 | I0005 | I0006 | I0007 | I0008 | I0009 | I0010 | I0011 | I0012 | I0013 | I0014 | I0015 | I0016 | I0017 | I0018 | I0019 | I0020 | I0021 | I0022 | I0023 | I0024 |
| --- | --- | --- | --- | --- | --- | --- | --- | --- | --- | --- | --- | --- | --- | --- | --- | --- | --- | --- | --- | --- | --- | --- | --- | --- | --- |
| RODS_bend_pick_up | I0001 |  |  |  |  |  |  |  |  |  |  |  |  |  |  |  |  |  |  |  |  |  |  |  |  |
| RODS_standing_long | I0002 | 0.014 |  |  |  |  |  |  |  |  |  |  |  |  |  |  |  |  |  |  |  |  |  |  |  |
| RODS_walk_up_stairs | I0003 | 0.072 | 0.078 |  |  |  |  |  |  |  |  |  |  |  |  |  |  |  |  |  |  |  |  |  |  |
| RODS_run | I0004 | 0.074 | 0.111 | 0.114 |  |  |  |  |  |  |  |  |  |  |  |  |  |  |  |  |  |  |  |  |  |
| RODS_outdoors_max_1km | I0005 | -0.01 | 0.12 | 0.172 | 0.119 |  |  |  |  |  |  |  |  |  |  |  |  |  |  |  |  |  |  |  |  |
| RODS_walk_avoid_obstacles | I0006 | 0.059 | 0.136 | 0.221 | 0.011 | 0.261 |  |  |  |  |  |  |  |  |  |  |  |  |  |  |  |  |  |  |  |
| RODS_dance | I0007 | 0.013 | 0.043 | 0.111 | 0.447 | 0.161 | 0.066 |  |  |  |  |  |  |  |  |  |  |  |  |  |  |  |  |  |  |
| RODS_public_transport | I0008 | -0.04 | 0.015 | 0.08 | 0.122 | 0.207 | 0.088 | 0.223 |  |  |  |  |  |  |  |  |  |  |  |  |  |  |  |  |  |
| RODS_turn_key_in_lock | I0009 | -0.01 | -0.091 | -0.083 | -0.178 | -0.2 | -0.17 | -0.241 | -0.169 |  |  |  |  |  |  |  |  |  |  |  |  |  |  |  |  |
| RODS_carry_put_down_heavy_object | I0010 | 0.056 | -0.042 | -0.169 | 0.02 | -0.032 | -0.151 | 0.019 | -0.043 | -0.019 |  |  |  |  |  |  |  |  |  |  |  |  |  |  |  |
| RODS_move_chair | I0011 | 0.021 | -0.112 | -0.056 | -0.079 | -0.156 | -0.135 | -0.089 | -0.099 | 0.038 | 0.053 |  |  |  |  |  |  |  |  |  |  |  |  |  |  |
| RODS_catch_object | I0012 | -0.07 | -0.141 | -0.103 | -0.159 | -0.088 | 0 | -0.15 | -0.102 | 0.051 | -0.069 | -0.039 |  |  |  |  |  |  |  |  |  |  |  |  |  |
| RODS_wash_upper_body | I0013 | -0.15 | -0.276 | -0.126 | -0.3 | -0.26 | -0.244 | -0.274 | -0.266 | 0.081 | -0.074 | 0.035 | 0.05 |  |  |  |  |  |  |  |  |  |  |  |  |
| RODS_wash_lower_body | I0014 | 0.023 | -0.153 | -0.062 | -0.18 | -0.248 | -0.137 | -0.193 | -0.188 | -0.004 | -0.021 | 0.001 | -0.122 | 0.448 |  |  |  |  |  |  |  |  |  |  |  |
| RODS_shower | I0015 | -0.01 | -0.071 | 0.013 | -0.114 | -0.05 | 0.002 | -0.122 | -0.059 | -0.167 | -0.108 | 0.014 | -0.132 | 0.259 | 0.325 |  |  |  |  |  |  |  |  |  |  |
| RODS_brush_teeth | I0016 | -0.09 | -0.177 | -0.143 | -0.236 | -0.176 | -0.069 | -0.182 | -0.114 | 0.187 | -0.051 | 0.008 | 0.087 | 0.206 | 0.036 | -0.01 |  |  |  |  |  |  |  |  |  |
| RODS_toilet | I0017 | 0.081 | -0.033 | 0.05 | -0.052 | -0.06 | 0.07 | -0.065 | -0.019 | -0.11 | -0.078 | 0.032 | -0.167 | -0.033 | 0.055 | 0.284 | -0.038 |  |  |  |  |  |  |  |  |
| RODS_dress_upper_body | I0018 | -0.16 | -0.198 | -0.113 | -0.234 | -0.215 | -0.111 | -0.228 | -0.189 | -0.007 | -0.069 | 0.033 | -0.059 | 0.377 | 0.217 | 0.332 | 0.094 | 0.193 |  |  |  |  |  |  |  |
| RODS_eat | I0019 | -0.13 | -0.078 | -0.13 | -0.174 | -0.141 | -0.088 | -0.181 | -0.128 | 0.036 | 0.05 | -0.075 | -0.04 | 0.022 | -0.04 | -0.112 | 0.047 | -0.06 | 0.098 |  |  |  |  |  |  |
| RODS_dishes | I0020 | -0.08 | -0.085 | -0.119 | -0.26 | -0.156 | -0.147 | -0.149 | -0.075 | 0.161 | -0.005 | 0.001 | -0.005 | 0.062 | 0.094 | -0.072 | 0.034 | -0.064 | 0.069 | 0.164 |  |  |  |  |  |
| RODS_sandwich | I0021 | -0.18 | -0.217 | -0.172 | -0.189 | -0.117 | -0.157 | -0.107 | -0.085 | 0.067 | -0.004 | -0.003 | 0.111 | 0.159 | -0.003 | -0.109 | 0.075 | -0.06 | 0.018 | 0.012 | 0.287 |  |  |  |  |
| RODS_shopping | I0022 | -0.01 | 0.151 | -0.014 | 0.073 | 0.168 | 0.039 | 0.002 | 0.201 | -0.148 | -0.053 | -0.055 | -0.111 | -0.251 | -0.147 | -0.006 | -0.139 | -0.045 | -0.118 | -0.093 | -0.011 | -0.093 |  |  |  |
| RODS_general_practitioner | I0023 | -0.05 | 0.066 | 0.064 | 0.009 | 0.127 | 0.087 | 0.095 | 0.235 | -0.168 | -0.048 | -0.1 | -0.111 | -0.269 | -0.072 | -0.006 | -0.138 | -0.03 | -0.193 | -0.067 | -0.07 | -0.085 | 0.381 |  |  |
| RODS_read | I0024 | -0.09 | -0.061 | -0.194 | -0.173 | -0.127 | -0.065 | -0.196 | -0.063 | 0.078 | -0.093 | -0.019 | 0.093 | -0.063 | -0.111 | -0.138 | 0.041 | -0.075 | -0.063 | 0.13 | -0.014 | -0.036 | -0.086 | -0.071 |  |
|  | ave | -0.03 |  |  |  |  |  |  |  |  |  |  |  |  |  |  |  |  |  |  |  |  |  |  |  |
| **Criterion cut point** | **ave + 0.2** | **0.168** |  |  |  |  |  |  |  |  |  |  |  |  |  |  |  |  |  |  |  |  |  |  |  |

**Supplementary Table 5: Local dependency full sample (n=1226)**

| Statement | Item | I0001 | I0002 | I0003 | I0004 | I0005 | I0006 | I0007 | I0008 | I0009 | I0010 | I0011 | I0012 | I0013 | I0014 | I0015 | I0016 | I0017 | I0018 | I0019 | I0020 | I0021 | I0022 | I0023 | I0024 |
| --- | --- | --- | --- | --- | --- | --- | --- | --- | --- | --- | --- | --- | --- | --- | --- | --- | --- | --- | --- | --- | --- | --- | --- | --- | --- |
| RODS_bend_pick_up | I0001 |  |  |  |  |  |  |  |  |  |  |  |  |  |  |  |  |  |  |  |  |  |  |  |  |
| RODS_standing_long | I0002 | 0.003 |  |  |  |  |  |  |  |  |  |  |  |  |  |  |  |  |  |  |  |  |  |  |  |
| RODS_walk_up_stairs | I0003 | 0.079 | 0.031 |  |  |  |  |  |  |  |  |  |  |  |  |  |  |  |  |  |  |  |  |  |  |
| RODS_run | I0004 | 0.038 | 0.044 | 0.095 |  |  |  |  |  |  |  |  |  |  |  |  |  |  |  |  |  |  |  |  |  |
| RODS_outdoors_max_1km | I0005 | -0.031 | 0.126 | 0.151 | 0.095 |  |  |  |  |  |  |  |  |  |  |  |  |  |  |  |  |  |  |  |  |
| RODS_walk_avoid_obstacles | I0006 | 0.052 | 0.082 | 0.231 | 0.008 | 0.211 |  |  |  |  |  |  |  |  |  |  |  |  |  |  |  |  |  |  |  |
| RODS_dance | I0007 | -0.028 | -0.011 | 0.07 | 0.252 | 0.099 | 0.039 |  |  |  |  |  |  |  |  |  |  |  |  |  |  |  |  |  |  |
| RODS_public_transport | I0008 | 0.003 | 0.051 | 0.086 | 0.104 | 0.158 | 0.12 | 0.12 |  |  |  |  |  |  |  |  |  |  |  |  |  |  |  |  |  |
| RODS_turn_key_in_lock | I0009 | -0.015 | -0.105 | -0.12 | -0.146 | -0.176 | -0.151 | -0.169 | -0.148 |  |  |  |  |  |  |  |  |  |  |  |  |  |  |  |  |
| RODS_carry_put_down_heavy_object | I0010 | 0.032 | -0.042 | -0.133 | -0.066 | -0.052 | -0.129 | -0.025 | -0.052 | -0.038 |  |  |  |  |  |  |  |  |  |  |  |  |  |  |  |
| RODS_move_chair | I0011 | 0.016 | -0.105 | -0.024 | -0.107 | -0.148 | -0.053 | -0.061 | -0.065 | 0.007 | 0.05 |  |  |  |  |  |  |  |  |  |  |  |  |  |  |
| RODS_catch_object | I0012 | -0.059 | -0.085 | -0.117 | -0.149 | -0.079 | -0.028 | -0.137 | -0.097 | 0.005 | -0.006 | -0.033 |  |  |  |  |  |  |  |  |  |  |  |  |  |
| RODS_wash_upper_body | I0013 | -0.148 | -0.236 | -0.16 | -0.222 | -0.211 | -0.232 | -0.174 | -0.257 | 0.073 | -0.04 | 0.049 | 0.006 |  |  |  |  |  |  |  |  |  |  |  |  |
| RODS_wash_lower_body | I0014 | -0.004 | -0.162 | -0.045 | -0.147 | -0.225 | -0.138 | -0.096 | -0.215 | -0.022 | -0.037 | 0.016 | -0.107 | 0.391 |  |  |  |  |  |  |  |  |  |  |  |
| RODS_shower | I0015 | -0.034 | -0.085 | 0.002 | -0.102 | -0.025 | -0.034 | -0.092 | -0.089 | -0.129 | -0.081 | 0.028 | -0.135 | 0.254 | 0.306 |  |  |  |  |  |  |  |  |  |  |
| RODS_brush_teeth | I0016 | -0.118 | -0.153 | -0.122 | -0.202 | -0.139 | -0.085 | -0.131 | -0.114 | 0.176 | -0.025 | -0.007 | 0.078 | 0.195 | 0.02 | -0.002 |  |  |  |  |  |  |  |  |  |
| RODS_toilet | I0017 | 0.066 | -0.034 | 0.057 | 0.003 | -0.052 | 0.045 | -0.071 | -0.062 | -0.07 | -0.078 | 0.036 | -0.164 | -0.03 | 0.063 | 0.19 | -0.044 |  |  |  |  |  |  |  |  |
| RODS_dress_upper_body | I0018 | -0.162 | -0.17 | -0.124 | -0.17 | -0.183 | -0.126 | -0.138 | -0.16 | 0.03 | -0.076 | -0.009 | -0.058 | 0.315 | 0.166 | 0.261 | 0.111 | 0.117 |  |  |  |  |  |  |  |
| RODS_eat | I0019 | -0.095 | -0.061 | -0.153 | -0.156 | -0.104 | -0.106 | -0.114 | -0.115 | 0.009 | 0.052 | -0.073 | -0.055 | 0.036 | -0.037 | -0.089 | 0.053 | -0.033 | 0.148 |  |  |  |  |  |  |
| RODS_dishes | I0020 | -0.061 | -0.089 | -0.102 | -0.197 | -0.139 | -0.123 | -0.078 | -0.104 | 0.132 | -0.005 | 0.019 | -0.014 | 0.036 | 0.09 | -0.027 | 0.07 | -0.031 | 0.075 | 0.115 |  |  |  |  |  |
| RODS_sandwich | I0021 | -0.164 | -0.202 | -0.176 | -0.144 | -0.141 | -0.168 | -0.056 | -0.097 | 0.07 | 0.024 | 0.02 | 0.064 | 0.142 | 0.005 | -0.128 | 0.09 | -0.071 | 0.02 | 0.026 | 0.309 |  |  |  |  |
| RODS_shopping | I0022 | -0.065 | 0.06 | -0.054 | 0.048 | 0.125 | 0.03 | 0.007 | 0.187 | -0.134 | -0.093 | -0.045 | -0.084 | -0.168 | -0.088 | -0.001 | -0.1 | -0.089 | -0.097 | -0.072 | -0.004 | -0.029 |  |  |  |
| RODS_general_practitioner | I0023 | -0.012 | 0.005 | 0.029 | 0.022 | 0.098 | 0.083 | 0.033 | 0.218 | -0.106 | -0.084 | -0.073 | -0.105 | -0.242 | -0.095 | -0.023 | -0.131 | -0.015 | -0.136 | -0.086 | -0.079 | -0.085 | 0.273 |  |  |
| RODS_read | I0024 | -0.081 | -0.04 | -0.188 | -0.201 | -0.142 | -0.084 | -0.151 | -0.057 | 0.098 | -0.093 | -0.054 | 0.111 | -0.057 | -0.082 | -0.113 | 0.01 | -0.079 | -0.045 | 0.102 | -0.042 | -0.034 | -0.04 | -0.058 |  |
|  | ave | -0.031 |  |  |  |  |  |  |  |  |  |  |  |  |  |  |  |  |  |  |  |  |  |  |  |
| **Criterion cut point** | **ave + 0.2** | **0.169** |  |  |  |  |  |  |  |  |  |  |  |  |  |  |  |  |  |  |  |  |  |  |  |

**Supplementary Table 6: DIF summary full sample (n=1226)**

|  |  | Country | Country | Sex | Sex | Agegroup | Agegroup |
| --- | --- | --- | --- | --- | --- | --- | --- |
|  |  | Uniform | Non-uniform | Uniform | Non-uniform | Uniform | Non-uniform |
| Item | Statement | Probability | Probability | Probability | Probability | Probability | Probability |
| 1 | RODS_bend_pick_up | 0.051 | 0.362 | 0.465 | 0.793 | 0.816 | 0.026 |
| 2 | RODS_standing_long | 0.000 | 0.000 | 0.198 | 0.480 | 0.941 | 0.033 |
| 3 | RODS_walk_up_stairs | 0.000 | 0.003 | 0.364 | 0.210 | 0.524 | 0.925 |
| 4 | RODS_run | 0.000 | 0.000 | 0.651 | 0.939 | 0.213 | 0.057 |
| 5 | RODS_outdoors_max_1km | 0.000 | 0.000 | 0.156 | 0.983 | 0.042 | 0.623 |
| 6 | RODS_walk_avoid_obstacles | 0.000 | 0.006 | 0.888 | 0.147 | 0.170 | 0.975 |
| 7 | RODS_dance | 0.090 | 0.384 | 0.982 | 0.140 | 0.990 | 0.091 |
| 8 | RODS_public_transport | 0.000 | 0.046 | 0.004 | 0.992 | 0.000 | 0.311 |
| 9 | RODS_turn_key_in_lock | 0.007 | 0.020 | 0.266 | 0.781 | 0.195 | 0.681 |
| 10 | RODS_carry_put_down_heavy_object | 0.000 | 0.478 | 0.850 | 0.419 | 0.836 | 0.000 |
| 11 | RODS_move_chair | 0.006 | 0.002 | 0.452 | 0.547 | 0.396 | 0.988 |
| 12 | RODS_catch_object | 0.000 | 0.023 | 0.807 | 0.029 | 0.006 | 0.000 |
| 13 | RODS_wash_upper_body | 0.000 | 0.001 | 0.333 | 0.195 | 0.008 | 0.180 |
| 14 | RODS_wash_lower_body | 0.000 | 0.371 | 0.097 | 0.125 | 0.009 | 0.700 |
| 15 | RODS_shower | 0.000 | 0.005 | 0.541 | 0.342 | 0.683 | 0.537 |
| 16 | RODS_brush_teeth | 0.000 | 0.884 | 0.094 | 0.928 | 0.521 | 0.444 |
| 17 | RODS_toilet | 0.000 | 0.111 | 0.027 | 0.689 | 0.334 | 0.610 |
| 18 | RODS_dress_upper_body | 0.520 | 0.048 | 0.022 | 0.917 | 0.583 | 0.224 |
| 19 | RODS_eat | 0.618 | 0.599 | 0.517 | 0.916 | 0.647 | 0.295 |
| 20 | RODS_dishes | 0.124 | 0.156 | 0.673 | 0.829 | 0.816 | 0.133 |
| 21 | RODS_sandwich | 0.000 | 0.302 | 0.620 | 0.927 | 0.450 | 0.663 |
| 22 | RODS_shopping | 0.007 | 0.004 | 0.033 | 0.930 | 0.045 | 0.975 |
| 23 | RODS_general_practitioner | 0.000 | 0.144 | 0.032 | 0.998 | 0.037 | 0.164 |
| 24 | RODS_read | 0.308 | 0.128 | 0.242 | 1.000 | 0.077 | 0.881 |
|  |  |  |  |  |  |  |  |

**Supplementary Table 7: Expected values for each item by country/region**

|  |  | Level | Bangladesh | NorthEUR | SouthEUR | NorthAmerica | Asia | Other | Overall |
| --- | --- | --- | --- | --- | --- | --- | --- | --- | --- |
| Item | Statement | [N] | [189] | [397] | [259] | [125] | [189] | [65] |  |
| 1 | RODS_bend_pick_up | Mean | 0.70 | 1.02 | 1.29 | 1.28 | 1.20 | 1.23 | 1.09 |
| 2 | RODS_standing_long | Mean | 0.48 | 0.43 | 0.87 | 0.66 | 1.17 | 0.86 | 0.67 |
| 3 | RODS_walk_up_stairs | Mean | 0.46 | 0.82 | 0.89 | 1.01 | 1.03 | 0.82 | 0.83 |
| 4 | RODS_run | Mean | 0.15 | 0.24 | 0.38 | 0.45 | 0.53 | 0.52 | 0.33 |
| 5 | RODS_outdoors_max_1km | Mean | 0.73 | 0.66 | 0.85 | 0.91 | 0.93 | 0.82 | 0.78 |
| 6 | RODS_walk_avoid_obstacles | Mean | 0.77 | 1.04 | 1.01 | 1.27 | 1.27 | 1.05 | 1.05 |
| 7 | RODS_dance | Mean | 0.36 | 0.41 | 0.54 | 0.61 | 0.61 | 0.78 | 0.52 |
| 8 | RODS_public_transport | Mean | 0.65 | 0.69 | 1.00 | 1.15 | 1.06 | 0.91 | 0.86 |
| 9 | RODS_turn_key_in_lock | Mean | 0.86 | 1.41 | 1.53 | 1.58 | 1.35 | 1.60 | 1.38 |
| 10 | RODS_carry_put_down_heavy_object | Mean | 0.47 | 0.76 | 1.06 | 0.85 | 0.83 | 0.91 | 0.80 |
| 11 | RODS_move_chair | Mean | 0.80 | 1.27 | 1.46 | 1.38 | 1.28 | 1.42 | 1.27 |
| 12 | RODS_catch_object | Mean | 1.43 | 1.33 | 1.48 | 1.38 | 1.19 | 1.53 | 1.38 |
| 13 | RODS_wash_upper_body | Mean | 0.95 | 1.60 | 1.56 | 1.67 | 1.44 | 1.67 | 1.50 |
| 14 | RODS_wash_lower_body | Mean | 0.80 | 1.34 | 1.44 | 1.45 | 1.34 | 1.49 | 1.31 |
| 15 | RODS_shower | Mean | 0.89 | 1.28 | 1.34 | 1.47 | 1.43 | 1.25 | 1.28 |
| 16 | RODS_brush_teeth | Mean | 1.41 | 1.70 | 1.66 | 1.80 | 1.47 | 1.77 | 1.64 |
| 17 | RODS_toilet | Mean | 0.91 | 1.43 | 1.48 | 1.61 | 1.52 | 1.49 | 1.41 |
| 18 | RODS_dress_upper_body | Mean | 1.13 | 1.51 | 1.59 | 1.63 | 1.53 | 1.67 | 1.50 |
| 19 | RODS_eat | Mean | 1.41 | 1.69 | 1.76 | 1.72 | 1.75 | 1.86 | 1.69 |
| 20 | RODS_dishes | Mean | 0.42 | 1.23 | 1.38 | 1.40 | 1.24 | 1.36 | 1.26 |
| 21 | RODS_sandwich | Mean | 0.67 | 1.49 | 1.49 | 1.55 | 1.09 | 1.64 | 1.45 |
| 22 | RODS_shopping | Mean | 0.63 | 0.82 | 1.08 | 1.12 | 1.13 | 1.02 | 0.93 |
| 23 | RODS_general_practitioner | Mean | 0.84 | 1.04 | 1.34 | 1.41 | 1.27 | 1.23 | 1.16 |
| 24 | RODS_read | Mean | 1.22 | 1.60 | 1.73 | 1.61 | 1.60 | 1.74 | 1.58 |

**Supplementary Figure 1: DIF-by-Geographic region plot for item 1 Bend forward and pick something up**

**
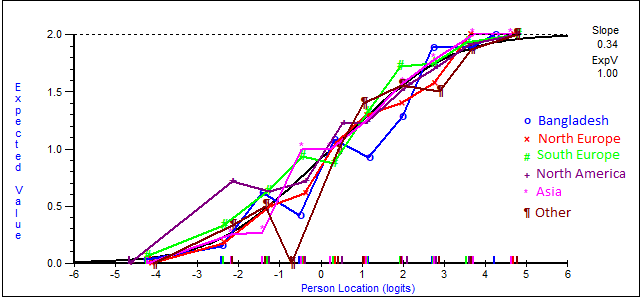
**

**Supplementary Figure 2: DIF-by-Geographic region plot for item 2 Remain standing for a long time period**
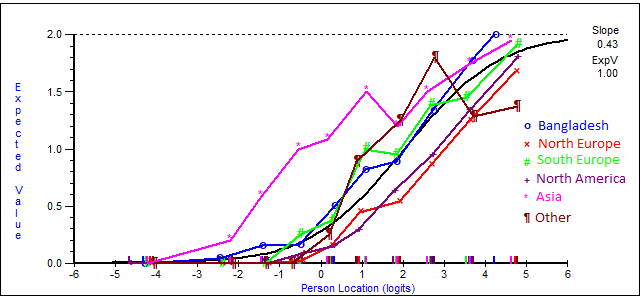


**Supplementary Figure 3: DIF-by-Geographic region plot for item 3 Walk up a flight of stairs**

**
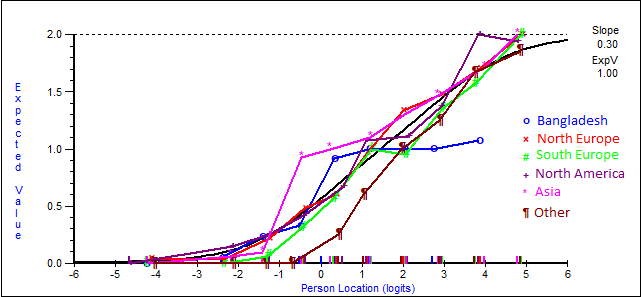
**

**Supplementary Figure 4: DIF-by-Geographic region plot for item 4 Run**

**
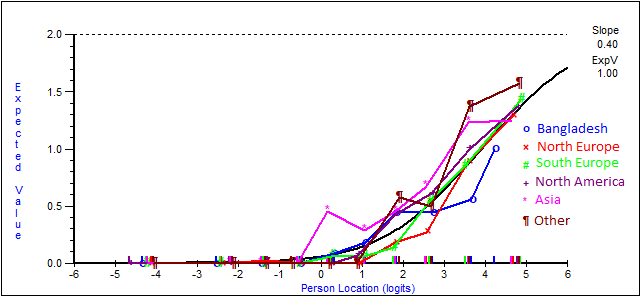
**

**Supplementary Figure 5: DIF-by-Geographic region plot for item 5 Walk outdoors, up to max 1 kilometre**

**
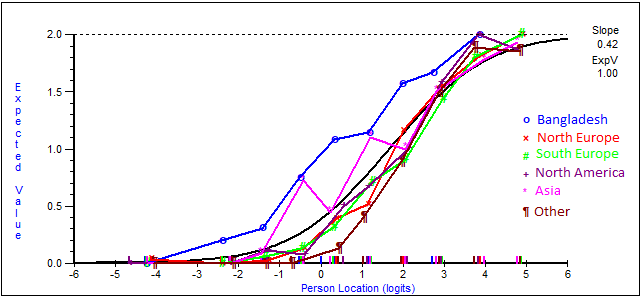
**

**Supplementary Figure 6: DIF-by-Geographic region plot for item 6 Walk while avoiding obstacles**

**
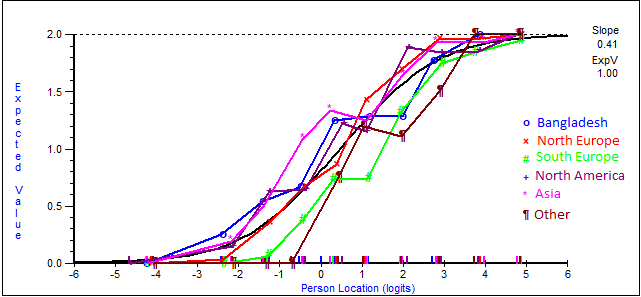
**

**Supplementary Figure 7: DIF-by-Geographic region plot for item 7 Dance**

**
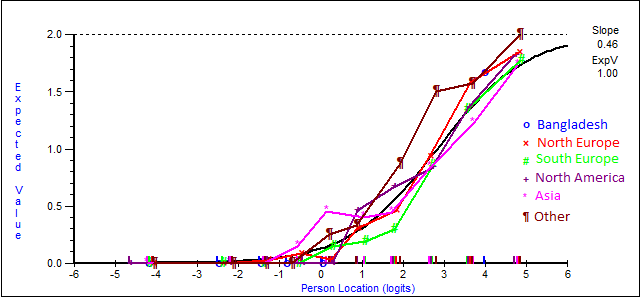
**

**Supplementary Figure 8: DIF-by-Geographic region plot for item 8 Travel by public transport**

**
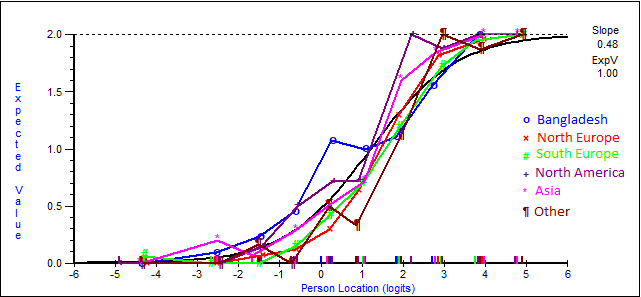
**

**Supplementary Figure 9: DIF-by-Geographic region plot for item 9 Turn a key in a lock**

**
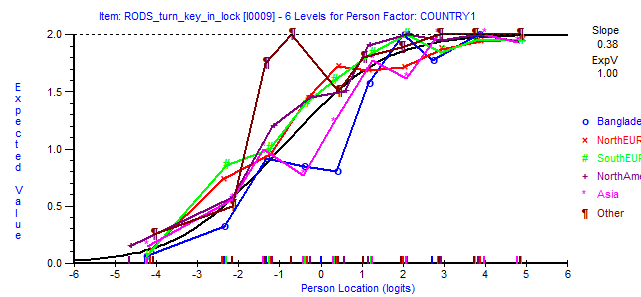
**

**Supplementary Figure 10: DIF-by-Geographic region plot for item 10 Carry and put down a heavy object**

**
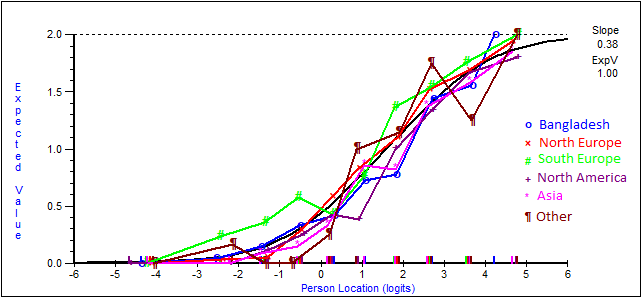
**

**Supplementary Figure 11: DIF-by-Geographic region plot for item 11 Move a chair**

**
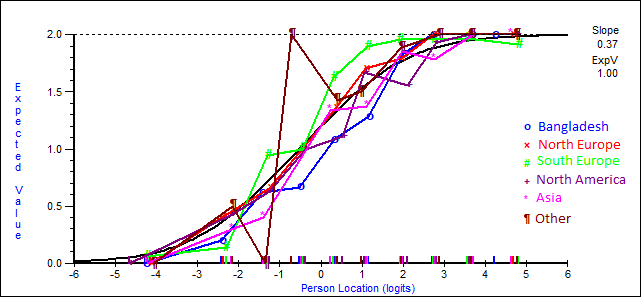
**

**Supplementary Figure 12: DIF-by-Geographic region plot for item 12 Catch an object (e.g. a ball)**

**
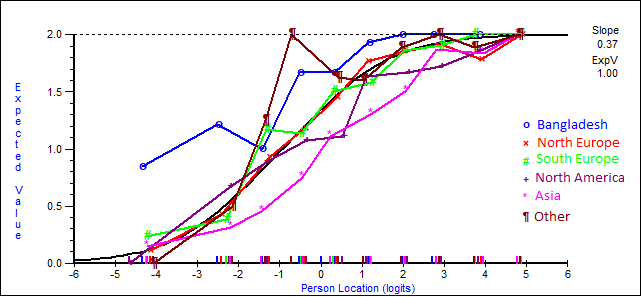
**

**Supplementary Figure 13: DIF-by-Geographic region plot for item 13 Wash your upper body**

**
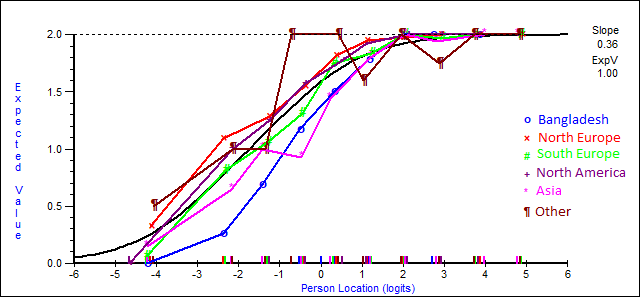
**

**Supplementary Figure 14: DIF-by-Geographic region plot for item 14 Wash your lower body**

**
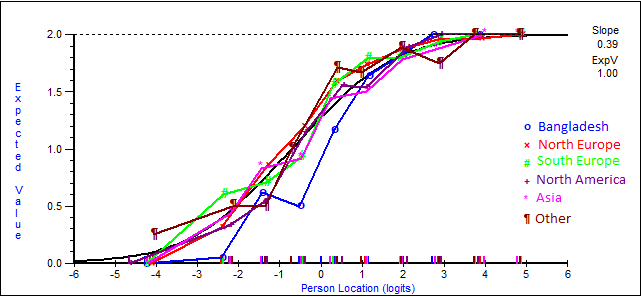
**

**Supplementary Figure 15: DIF-by-Geographic region plot for item 15 Take a shower**

**
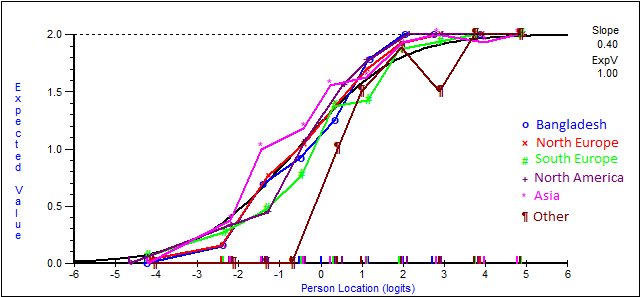
**

**Supplementary Figure 16: DIF-by-Geographic region plot for item 16 Brush your teeth**

**
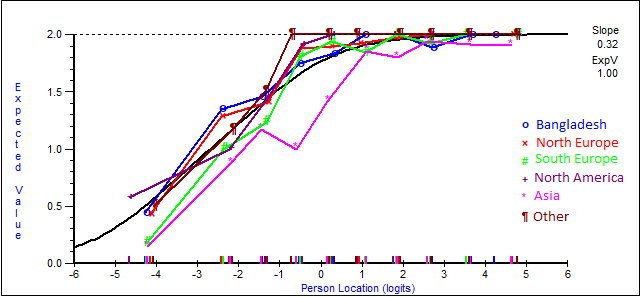
**

**Supplementary Figure 17: DIF-by-Geographic region plot for item 17 Sit on/go to a toilet**

**
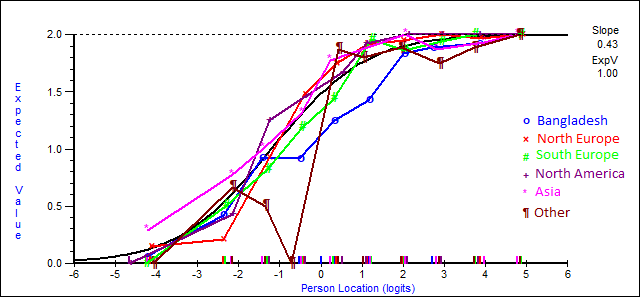
**

**Supplementary Figure 18: DIF-by-Geographic region plot for item 18 Dress your upper body**

**
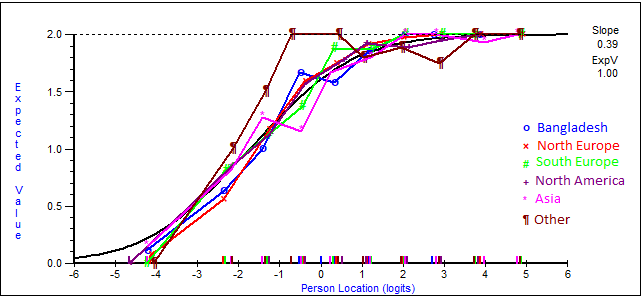
**

**Supplementary Figure 19: DIF-by-Geographic region plot for item 19 Eat**

**
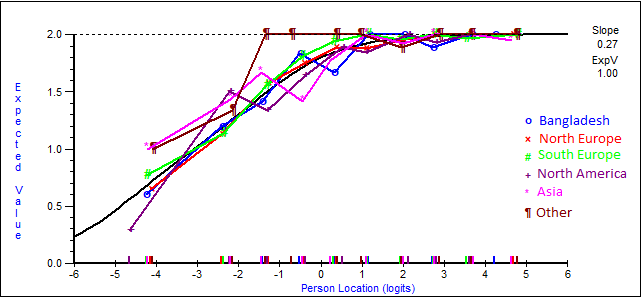
**

**Supplementary Figure 20: DIF-by-Geographic region plot for item 20 Do the dishes**

**
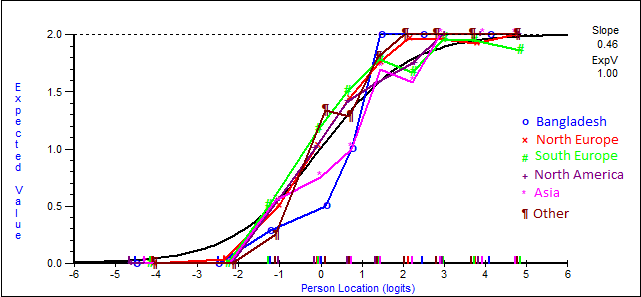
**

**Supplementary Figure 21: DIF-by-Geographic region plot for item 21 Make a sandwich**

**
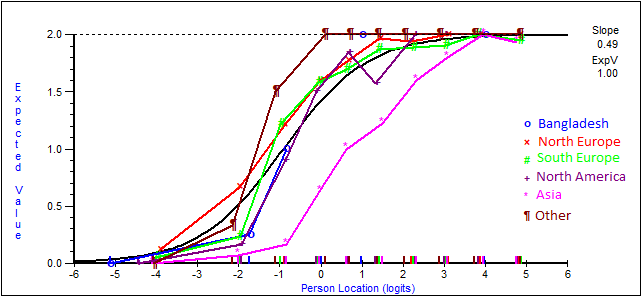
**

**Supplementary Figure 22: DIF-by-Geographic region plot for item 22 Do the shopping**

**
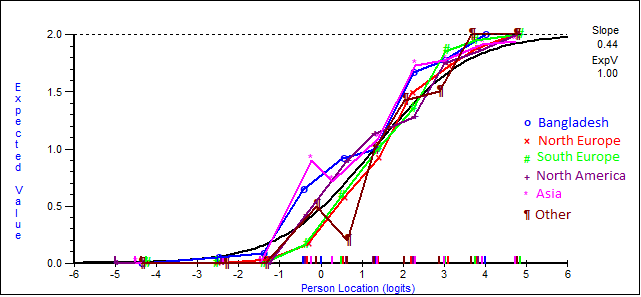
**

**Supplementary Figure 23: DIF-by-Geographic region plot for item 23 Go to the general practitioner**

**
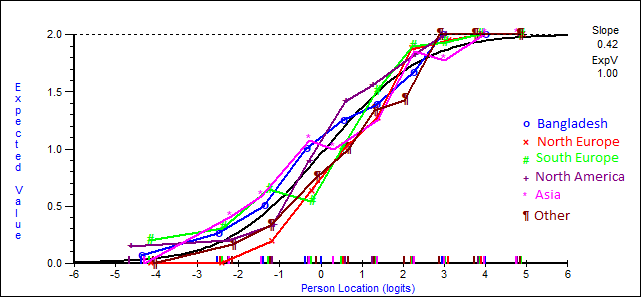
**

**Supplementary Figure 24: DIF-by-Geographic region plot for item 24 Read a book/newspaper**

**
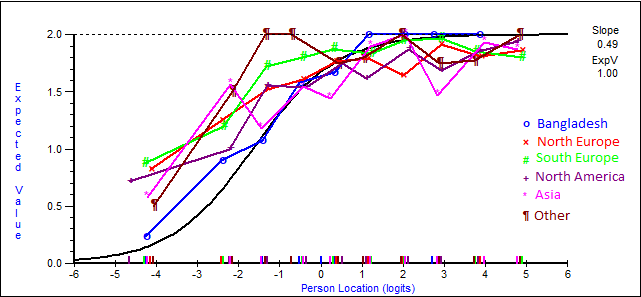
**
